# Cross-Kingdom Antagonistic Interactions Between Environmental Antibiotic-Resistant Bacteria and Yeasts in Pastoral Ecosystems

**DOI:** 10.3390/microorganisms14050991

**Published:** 2026-04-28

**Authors:** Alper Melih Ucar, Deividas Paliovkinas, Živilė Strazdaitė-Žielienė, Modestas Petrauskas, Elena Servienė, Juliana Lukša-Žebelovič

**Affiliations:** 1 Laboratory of Genetics, State Scientific Research Institute Nature Research Centre, Akademijos Str. 2, LT-08412 Vilnius, Lithuania; 2 Department of Chemistry and Bioengineering, Faculty of Fundamental Sciences, Vilnius Gediminas Technical University (VILNIUS TECH), Saulėtekio al. 11, LT-10223 Vilnius, Lithuania

**Keywords:** antibiotic-resistant bacteria, agricultural ecosystems, antagonistic yeast, microbial interactions, biocontrol yeast

## Abstract

Pasture-based farming systems remain understudied as a source of antibiotic-resistant bacteria and antagonistic yeasts under low-input management. In this study, soil, feed, and freshwater samples from Lithuanian farms were analyzed to characterize resistance phenotypes and inhibitory interactions among cultivable microorganisms. Bacterial and yeast isolates, representing 14 and 9 genera, respectively, were identified using molecular methods. Bacterial isolates recovered under antibiotic-selective conditions exhibited resistance to at least one of four antibiotics (ampicillin, streptomycin, tetracycline, chloramphenicol), with 41% showing multidrug resistance and the highest frequencies observed for ampicillin (59%) and streptomycin (48%). Microbial distribution was habitat-specific, with soil containing the highest diversity of antibiotic-resistant bacteria and feed samples harboring the greatest abundance of yeasts. Functional assays demonstrated consistent antagonistic activity of several *Wickerhamomyces anomalus* strains against susceptible yeasts and antibiotic-resistant *Bacillus* spp. These findings highlight the coexistence of resistance and antagonistic traits in pasture-associated microbiota and suggest their potential interaction under controlled conditions.

## 1. Introduction

Pasture-based farming systems comprise complex, interconnected microbial habitats in which soil, feed, and freshwater compartments support diverse, interacting microbial populations. These environments are continuously exposed to biological inputs from plants, animals, and surrounding landscapes, creating conditions that favor both microbial persistence and competition [1,2]. Although agricultural microbiomes are increasingly investigated using high-throughput sequencing approaches, experimental methods that rely on viable isolates remain essential for characterizing phenotypic traits, such as antibiotic resistance and antagonistic activity, that cannot be inferred solely from sequence data [3].

Antibiotic-resistant bacteria (ARB) are widely detected in agricultural soils, water bodies, and animal-associated substrates, even in settings where antibiotic residues are minimal or undetectable. Research conducted in Lithuania has demonstrated that soil and sediment microbiota may harbor resistance determinants despite limited contemporary antimicrobial exposure [4,5]. Surveys of farmland environments frequently report multidrug-resistant isolates belonging to genera such as *Pseudomonas*, *Acinetobacter*, *Stenotrophomonas*, *Sphingobacterium*, *Bacillus*, and members of the *Enterobacteriaceae* [6,7,8]. Resistance in environmental bacteria may arise from intrinsic genetic traits, historical exposure to antimicrobial compounds, co-selection driven by metal or biocide exposure, or horizontal gene transfer within complex microbial communities [9,10]. In dairy grazing systems, animals continuously ingest soil particles, plant-associated microbiota, and waterborne microorganisms. ARB in pastoral systems circulate across environmental compartments and livestock, with transmission occurring through ingestion and subsequent excretion via the gastrointestinal tract into receiving environments [11]. This process may increase the risk of multidrug resistance in livestock and complicate disease control. In addition to livestock, other animals such as birds and small mammals may contribute to the redistribution of microorganisms between environmental compartments, further supporting microbial exchange [12]. These interactions create a dynamic system in which microorganisms are continuously transferred, reshaped, and reintroduced across habitats [13]. Culture-based isolation on antibiotic-supplemented media provides a functional means of recovering bacteria capable of growth under defined selective conditions and allows subsequent phenotypic characterization of resistance profiles. While such approaches do not quantify environmental prevalence, they enable direct assessment of resistance phenotypes in viable isolates [14].

In parallel, the same agricultural niches harbor diverse yeast communities that actively participate in microbial competition for nutrients and space [15]. Environmental yeasts are common inhabitants of plant surfaces and in nutrient-rich substrates such as animal feed [16]. To maintain a competitive advantage, many species have evolved to produce inhibitory secondary metabolites or killer toxins (mycocins) that suppress competing fungi and, in some cases, bacteria [17]. These antagonistic properties have been extensively investigated in the context of postharvest biocontrol and plant pathogen suppression [18]. Species such as *Wickerhamomyces anomalus*, *Aureobasidium pullulans*, *Debaryomyces hansenii*, and *Pichia kudriavzevii* have been reported in soil- and plant-associated environments and are known to exhibit antimicrobial activity under laboratory conditions [19].

Despite substantial research on environmental antibiotic resistance and yeast-mediated antagonism as independent domains, their functional intersection within pastoral agricultural systems remains insufficiently characterized [20]. Studies addressing environmental antibiotic resistance have primarily focused on the occurrence of resistant bacteria, resistance genes, and their environmental drivers, whereas investigations of antagonistic yeasts have largely examined their activity against plant pathogens or laboratory model organisms [21,22]. Limited attention has been given to the recovery and comparative analysis of phenotypically resistant bacteria and antagonistic yeasts from the same habitat compartments. Microbial interactions between yeast and bacteria impose selective pressures on microbial dynamics, potentially influencing resistance phenotypes and the dissemination of antimicrobial resistance (AMR) [23]. However, most studies evaluating yeast antibacterial activity have focused on clinically relevant pathogens or laboratory model strains rather than environmentally derived antibiotic-resistant isolates [24,25]. Unlike well-studied biocontrol applications on clinically relevant strains, the extent to which the prevalence of farmland communities is influenced by the extent of antagonistic activity remains unclear. Understanding which resistant bacteria and antagonistic yeasts can be recovered from pasture-associated compartments provides insight into potential microbial interactions.

Pasture-associated environments represent complex microbial habitats where selective pressures may shape both antibiotic resistance and cross-kingdom interactions, yet these components are rarely investigated in an integrated manner. Based on this, the aim of the study was to investigate the occurrence of antibiotic-resistant bacteria and yeasts in interconnected pasture-associated environments (soil, feed, and freshwater) and to assess their potential interactions. We hypothesized that pasture-associated environments harbor distinct communities of cultivable antibiotic-resistant bacteria and antagonistic yeasts, and that antagonistic features-possessing yeasts exhibit inhibitory activity against antibiotic-resistant bacterial isolates, reflecting potential cross-kingdom interactions within these habitats. Bacterial isolates obtained under antibiotic-selective conditions were characterized using minimum inhibitory concentrations (MIC) testing, while yeast isolates were evaluated for antagonistic activity against sensitive yeasts and antibiotic-resistant bacterial strains. The findings reveal a preliminary phenotypic exploration of potential cross-kingdom interactions within agricultural niches, offering insight into how the antagonistic properties of environmental yeasts may influence the proliferation and persistence of antibiotic-resistant bacteria. A comprehensive understanding of phenotypic interactions is essential for effective environmental monitoring. The distribution and composition of these communities serve as a critical indicator of environmental health and potential hazards to both animals and humans.

## 2. Materials and Methods

### 2.1. Sampling Design and Microbial Isolation

Samples were collected during June–August 2023 at three privately owned pasture-based dairy farms in Purvėnai, Molėtai, and Ignalina, Lithuania. At each farm, three subsamples of soil, feed, and freshwater were collected from grazing areas and processed independently for microbial isolation. In these areas, grass and hay represent typical on-site cow feed. Freshwater was sampled from the shorelines of waterbodies (0–10 cm depth) located in the farmland. Soil samples (0–10 cm depth) were collected using random sampling points in the grazing area. Soil and feed were collected into sterile bags, and freshwater into sterile 3 L bottles using aseptic techniques. Samples were transported under cooled conditions within 2 h and processed immediately or after short-term storage at 4 °C.

For bacterial isolation, soil and feed samples (200 g) were suspended in 1 L sterile distilled water, shaken for 1 h at room temperature, sequentially filtered (using 2 mm, 1 mm, and 0.05 mm), and centrifuged at 3000× *g* for 15 min. Freshwater samples (1 L) were filtered through a sieve (0.05 mm) and centrifuged identically. Aliquots (12 mL per sample) were centrifuged again at 3000× *g* for 15 min, discarded, and the pellets were pooled and resuspended in liquid LB to a final volume of 500 μL. Suspensions (100 μL) were plated on LB agar (1% peptone, 0.5%, yeast extract, 1% NaCl, 2% agar) supplemented with ampicillin (100 μg/mL), streptomycin (50 μg/mL), tetracycline (10 μg/mL), or chloramphenicol (25 μg/mL) (AppliChem, Darmstadt, Germany) and incubated overnight at 25 °C.

For yeast isolation, soil samples (200 g) were suspended in 50 mL of minimal dextrose medium (MD) (2% glucose, 1% (NH_4_)_2_SO_4_, 0.09% KH_2_PO_4_, 0.05% MgSO_4_, 0.023% K_2_HPO_4_, 0.01% NaCl, 0.01% CaCl_2_) and shaken for 15 min at 22 °C, while feed samples (200 g) were suspended in 2 L sterile distilled water and shaken for 1 h at 22 °C. Suspensions (12 mL), including freshwater samples, were centrifuged at 3000× *g* for 1 min, and 100 μL of the resuspended pellet was plated on YEPD agar (1% yeast extract, 2% peptone, 2% glucose, 2% agar) supplemented with chloramphenicol (50 μg/mL). Plates were incubated at 22 °C for 2–3 d.

Following incubation, morphologically distinct bacterial and yeast colonies were examined microscopically and subcultured on fresh LB or YEPD agar plates. Obtained pure isolates were incubated in liquid media and stored in 20% (*v*/*v*) glycerol at −70 °C.

### 2.2. Identification of Isolated Bacteria and Yeasts

Molecular identification of bacterial and yeast isolates was performed by PCR amplification of marker genes, followed by DNA preparation using different approaches for bacteria and yeasts.

For bacterial isolates, DNA was obtained by direct colony PCR. A single fresh colony was suspended in 5 μL of nuclease-free water and used directly as a template without prior DNA extraction. The 16S rRNA gene was amplified using universal bacterial primers W001 (5′-AGAGTTTGATCMTGGTCT-3′) and W002 (5′-GNTACCTTGTTACGACTT-3′). For yeast isolates, genomic DNA was extracted with the Genomic DNA Purification Kit (Thermo Fisher Scientific Baltics, Vilnius, Lithuania) according to the manufacturer’s instructions. The extracted DNA was used as a template for PCR amplification of the internal transcribed spacer (ITS) region using primers ITS1 (5′-TCCGTAGGTGAACCTGCGG-3′) and ITS4 (5′-TCCTCCGCTTATTGATATGC-3′).

PCR reactions (100 μL) were performed using DreamTaq polymerase (Thermo Fisher Scientific Baltics, Vilnius, Lithuania) under standard conditions: 10 μL DreamTaq 10× Buffer, 10 μL of 2 mM dNTP Mix, 1 μL of each primer (10 μmol/L), 1 μL DreamTaq DNA Polymerase (5 U/μL), 1 μL DNA template, and nuclease-free water. Cycling parameters were as follows: for bacteria, 95 °C for 5 min; 30 cycles of 94 °C for 30 s, 45 °C for 30 s, and 72 °C for 2 min; and a final extension at 72 °C for 10 min. For yeasts, cycling consisted of 94 °C for 5 min; 25 cycles of 94 °C for 1 min, 53 °C for 30 s, and 72 °C for 30 s; followed by 72 °C for 10 min.

PCR products were verified by 1% agarose gel electrophoresis using GeneRuler DNA Ladder Mix (Thermo Fisher Scientific Baltics, Lithuania). For preliminary differentiation of isolates, amplicons were digested with FastDigest HinfI and HhaI restriction enzymes, and restriction profiles were analyzed on 2% agarose gels. Representative isolates showing distinct RFLP patterns were selected for sequencing.

Purified PCR products (GeneJet PCR Purification Kit, Thermo Fisher Scientific Baltics, Lithuania) were sequenced by BaseClear (Leiden, The Netherlands) using Sanger sequencing. Resulting sequences were compared to reference sequences BLAST+ v2.17.0 (EMBL-EBI database, accessed on 30 January 2026), and isolates showing >97% similarity to reference sequences were assigned to the corresponding genus and/or species.

### 2.3. Minimum Inhibitory Concentration (MIC) Assay

MICs for ampicillin, streptomycin, tetracycline, and chloramphenicol were determined for the 44 bacterial isolates using Liofilchem MIC Test Strips (Liofilchem, Roseto degli Abruzzi, Italy) following the manufacturer’s instructions. Each isolate was tested in triplicate (two technical replicates from the same inoculum and one independent biological replicate from a separately subcultured colony). The mean MIC values were interpreted according to the M100 Performance Standards for Antimicrobial Susceptibility Testing breakpoints established by the Clinical and Laboratory Standards Institute (CLSI). As standardized breakpoints for environmental isolates are limited, genera lacking CLSI-specific criteria were assigned based on those defined for phylogenetically or physiologically related taxa [14,26]. All MIC interpretative criteria, including susceptible (S), intermediate (I), and resistant (R) thresholds, are provided in Table S1.

### 2.4. Statistical Analysis of ARB in Pastoral Habitats

To assess whether the distribution of bacterial genera and resistance phenotypes differed significantly, a chi-square test of independence was applied. The analysis evaluated associations between bacterial genera and two categorical variables: habitat (soil, feed, freshwater) and resistance classification (resistant, susceptible), testing the null hypothesis that these variables are independent. Separate tests were performed for genus-habitat and genus-resistance pairs. For each test, resistance and habitat categories were converted into binary presence/absence data to generate contingency tables. Isolates classified as “intermediate” were grouped with resistant strains. Statistical significance was determined using a threshold of *p* < 0.05.

Correspondence analysis (CA) was performed using a genus-by-habitat contingency table to visualize associations between microbial taxa and environmental compartments. The ordination is based on chi-square distances calculated from genus-level isolate counts across habitats, with proximity between points indicating stronger associations between taxa and environmental compartments.

Resistance patterns were analyzed using a Jaccard similarity matrix based on a binary table in which rows represent genera and columns represent the four antibiotics. This metric was selected for its suitability with presence/absence data and its emphasis on shared resistance traits.

All statistical analyses and visualizations were conducted in Python v3.12.12 using numpy v2.0.2 [27], pandas v2.2.2 [28], scipy v1.16.3 [29], matplotlib v3.10.0 [30], seaborn v0.13.2 [31], and prince v0.16.3 [32].

### 2.5. Assessment of Antimicrobial Activity of Yeast Isolates

The antimicrobial activity of yeast isolates was evaluated using an inhibition assay. For testing yeast antagonistic activity, methylene blue (MB) agar plates (0.5% yeast extract, 0.5% peptone, 4% glucose, 1.05% citric acid, 3.53% Na_2_HPO_4_ × 12H_2_O, 2.5% agar, 0.002% methylene blue) adjusted to pH 4.0, 4.8, and 5.6, was inoculated with indicator yeast cells. Fresh yeast colonies were suspended in YEPD medium, and 5 μL aliquots were spotted onto the agar surface. Plates were incubated at 25 °C for 48 h, and inhibition zones surrounding the colonies were recorded. Strains producing reproducible inhibition zones were selected for further characterization. Inhibition halo diameter was measured five times from different edges of the same halo using ImageJ v1.54g [33], and the mean value was used for analysis.

For yeast–bacteria assays, bacterial indicator strains were suspended in sterile water and seeded into molten LB agar (pH 5.6), which was poured into Petri dishes. Yeast suspensions (5 μL) were spotted onto solidified LB agar and incubated at 30 °C for 48 h. Inhibition zones were assessed as described above.

## 3. Results and Discussion

### 3.1. Distribution and Habitat Associations of Bacterial and Yeast Isolates in Farmland-Associated Environments

**Figure 1:**
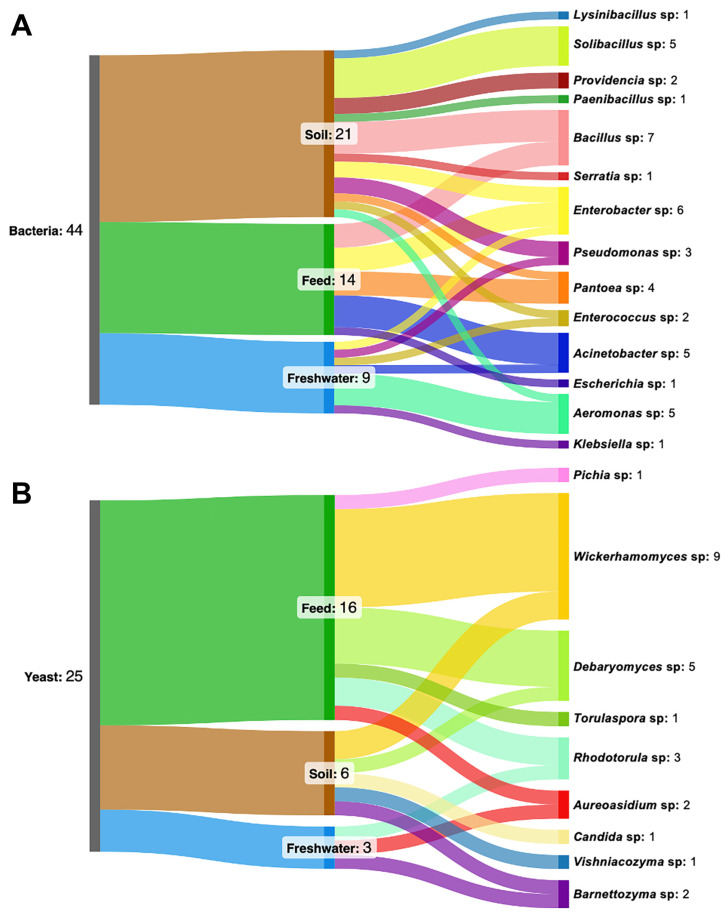
Distribution of antibiotic-resistant bacterial (**A**) and yeast (**B**) genera in farmland ecosystems. The left blocks represent the total number of isolates (bacteria, *n* = 44; yeast, *n* = 25), the middle blocks indicate habitat origin (soil, feed, freshwater), and the right blocks represent identified genera. Colors distinguish different habitats and taxonomic groups, with flows linking habitats to corresponding genera. The width of each flow is proportional to the number of isolates assigned to a given genus within each habitat, reflecting their relative abundance. The diagrams were generated using SankeyMATIC v1.2.0.

To explore potential interactions between environmental yeasts and antibiotic-resistant bacteria, bacterial isolates were first characterized for taxonomic composition and resistance phenotypes, after which yeast antagonistic activity against these isolates was assessed. In total, 69 morphologically distinct microbial isolates were obtained from soil, feed, and freshwater samples using culture-based methods. Bacterial isolates (*n* = 44) were obtained from antibiotic-supplemented media designed to suppress susceptible background microbiota and recover phenotypically resistant strains under selective pressure. In parallel, 25 yeast isolates were recovered from the same environments. Based on sequence similarity analysis, the bacterial isolates were assigned to 14 genera and the yeast isolates to 9 genera (Figure 1). Representative colonies of microbial isolates are presented in Figure S1.

The distribution of both bacteria and yeast was uneven across sampled habitats. Bacterial isolates were distributed across all three habitats, with soil yielding the highest number of ARB isolates (21 of 44) and the highest genus-level diversity (11 genera), followed by feed (14 isolates, 5 genera) and freshwater (9 isolates, 6 genera). *Bacillus* (16% of total isolates) and *Enterobacter* (14%) were the most frequently detected bacterial genera overall. Yeast isolates were most numerous and diverse in feed (16 isolates, 6 genera), followed by soil (6 isolates, 5 genera), and freshwater (3 isolates, 3 genera). Among yeast, *Wickerhamomyces* was the most frequently recovered yeast genus (36% of total isolates), followed by *Debaryomyces* (20%) and *Rhodotorula* (12%). These patterns reflect ecological differences among sampled habitats and selective pressures shaping microbial communities. Soil environments are known to support high microbial diversity and complex competitive interactions because of their heterogeneous structure and nutrient gradients [34].

In contrast, feed represents a dynamic substrate shaped by plant origin, storage conditions, and periodic renewal [35,36] conditions that favor rapid microbial colonization by both bacteria and yeast. Freshwater habitats contained fewer isolates and genera, which may reflect lower nutrient availability and continuous hydrological turnover that limit stable microbial establishment [37,38]. *Enterobacter* was the only genus recovered from all sampled habitats, consistent with the ubiquity of Enterobacteriaceae in cattle farm environments, where they circulate through manure, soil, and water [39].

**Figure 2:**
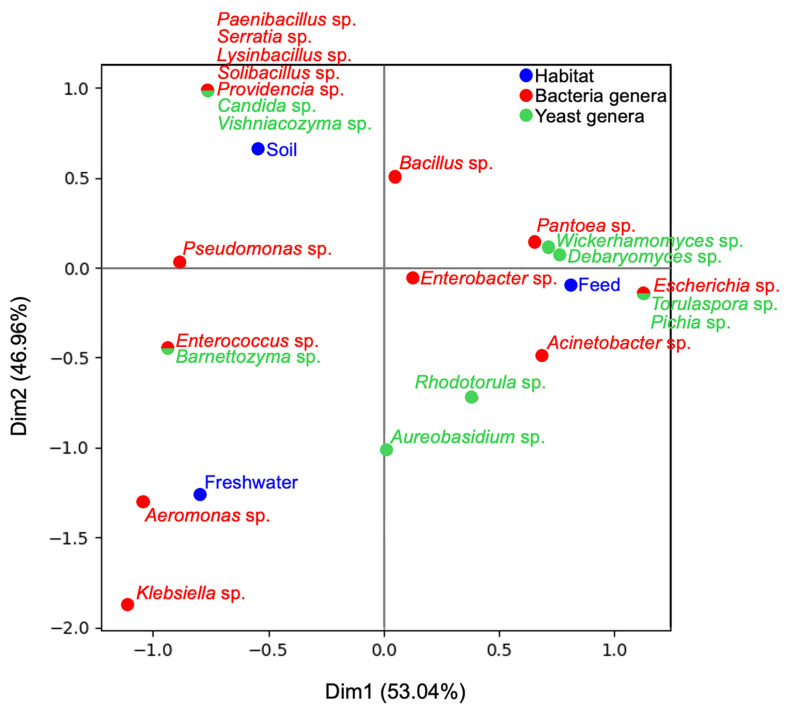
Correspondence analysis showing the distribution between antibiotic-resistant bacterial genera (red), yeast genera (green), and farmland habitat types (blue) based on genus-level isolate counts.

Correspondence analysis (CA) of genus-level isolate counts across habitats revealed structured distribution patterns (Figure 2). The first axis explained 53.04% of the total variation, and the second axis 46.96%.

Soil-derived microorganisms formed a distinct cluster along the positive Dim2 axis, and included several bacterial genera not recovered from other habitats, such as *Paenibacillus*, *Serratia*, *Lysinibacillus*, *Solibacillus*, and *Providencia*, along with yeast genera *Candida* and *Vishniacozyma*. The association of these taxa with soil cluster reflects the high habitat heterogeneity and niche specialization typical of soil microbial communities [40]. Members of *Paenibacillus* and *Lysinibacillus* are widely reported from soil and rhizosphere environments, often participating in plant growth-promotion and nutrient cycling [41,42]. Previous studies show that *Candida* and *Rhodotorula* frequently occur in soil-associated yeast communities, and that individual soil sites often harbor distinct yeast assemblages. *Vishniacozyma* is also commonly associated with plants, litter, and soils, supporting its proximity to the soil cluster in the present CA results [43,44].

Feed sample isolates clustered near the positive Dim1 axis, with associated genera including *Pantoea*, *Escherichia*, and *Acinetobacter*. Recently, antimicrobial-resistant strains from these genera have also been reported in farmland feed and other associated environments [45,46,47].

Yeast genera associated with feed included *Wickerhamomyces*, *Debaryomyces*, *Pichia*, and *Torulaspora*. The tight association of *Wickerhamomyces* and *Debaryomyces* to feed aligns with reports showing that forage and feed materials serve as major sources for plant-surface-associated yeasts [48].

Freshwater isolates formed a distinct cluster along the negative Dim2 axis, with *Aeromonas* and *Klebsiella* strongly associated, both genera known for their aquatic environmental persistence [49,50]. Yeast genera found in freshwater included *Rhodotorula*, *Barnettozyma*, and *Aureobasidium,* although they showed a different clustering pattern in the CA ordination. Pigmented yeasts, such as *Rhodotorula*, are frequently recovered from aquatic environments, while genera such as *Aureobasidium* and *Barnettozyma* have also been reported from water-associated habitats [51,52]. In pastoral systems, water sources are often subject to microbial inputs from surrounding soil, plant material, and livestock [53]. However, the taxa identified were primarily associated with aquatic environments and lacked terrestrial microbial inputs that would influence the recovered community.

### 3.2. Antibiotic-Resistance Patterns of Farmland-Associated Bacteria

**Figure 3:**
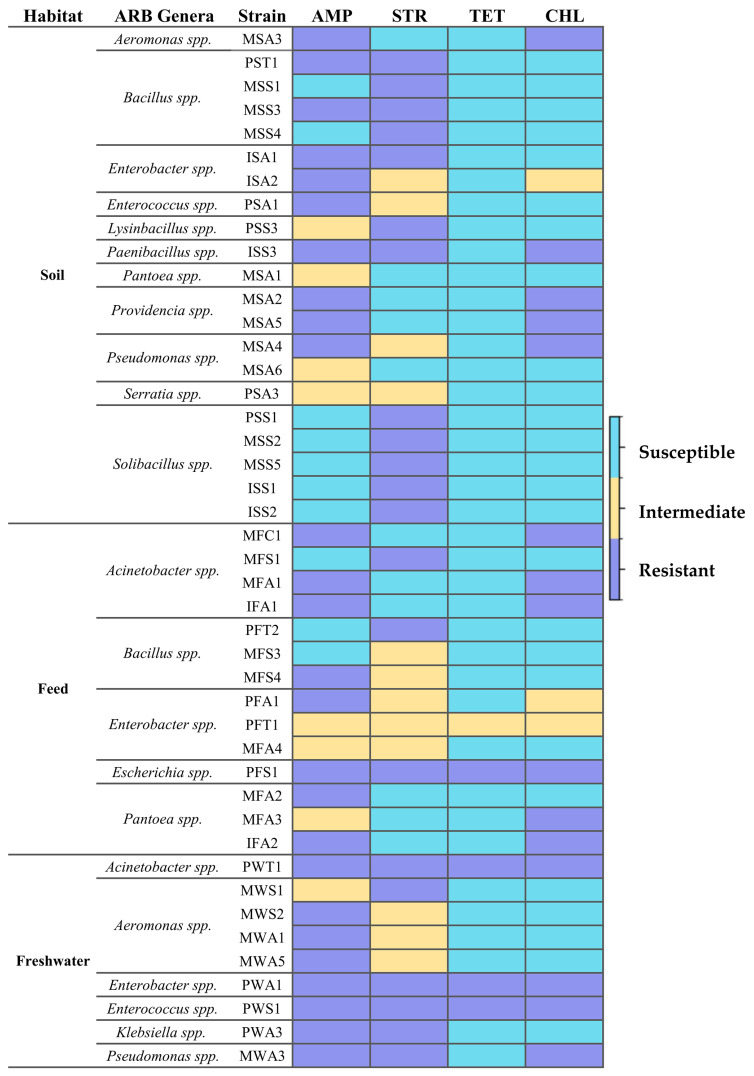
Heatmap of antibiotic resistance profiles of farmland-associated bacterial isolates based on MIC assay. MIC assay was conducted using ampicillin (AMP), streptomycin (STR), tetracycline (TET), and chloramphenicol (CHL) test strips. Strains were classified as susceptible (blue), intermediate (yellow), and resistant (purple) according to CLSI interpretive criteria for MIC values.

The 44 bacterial isolates recovered under antibiotic-selective conditions were subjected to MIC testing using gradient diffusion strips for ampicillin, streptomycin, tetracycline, and chloramphenicol. These antibiotics represent four distinct antimicrobial classes: β-lactam (ampicillin), aminoglycoside (streptomycin), tetracyclines (tetracycline), and phenicols (chloramphenicol). They were selected to provide broad coverage of major antibiotic mechanisms and to enable comparative assessment of phenotypic resistance across functionally diverse compounds. In addition, these antibiotics have been widely used in both clinical and agricultural settings and are commonly included in environmental antimicrobial resistance studies, allowing comparison with previously reported resistance patterns [54]. The phenotypic resistance profiles are summarized in Figure 3.

Resistance to ampicillin and streptomycin was the most common phenotype among the recovered isolates. Overall, 59% of strains were classified as resistant to ampicillin and 48% to streptomycin, whereas resistance to chloramphenicol was detected in 34% of isolates. According to the referenced standard, 18.2% of the isolates were classified as intermediate to ampicillin, 27.3% to streptomycin, and 6.8% to chloramphenicol. Tetracycline resistance was comparatively rare, occurring in four strains. Multidrug resistance was detected in 41% of the isolates. Resistance phenotypes varied substantially among isolates within the same genus except *Solibacillus* strains (PSS1, MSS2, MSS5, ISS1, and ISS2), which showed resistance exclusively to streptomycin. *Escherichia* PFS1 isolated from feed, *Acinetobacter* PWT1, *Enterobacter* PWA1, and *Enterococcus* PWS1 isolated from freshwater were resistant to all four antibiotics tested. Resistance to three antibiotics was found in *Paenibacillus* ISS3, and *Pseudomonas* MWA3 strains exhibited resistance to ampicillin, streptomycin, and chloramphenicol.

The association between bacterial genus and resistance phenotype was evaluated using chi-square analysis. Significant associations were observed for ampicillin (*p* = 0.0105), streptomycin (*p* = 0.0051), and chloramphenicol (*p* = 0.0482), whereas no statistically significant association was detected for tetracycline (*p* = 0.1631), likely due to its low prevalence in this sample set.

**Figure 4:**
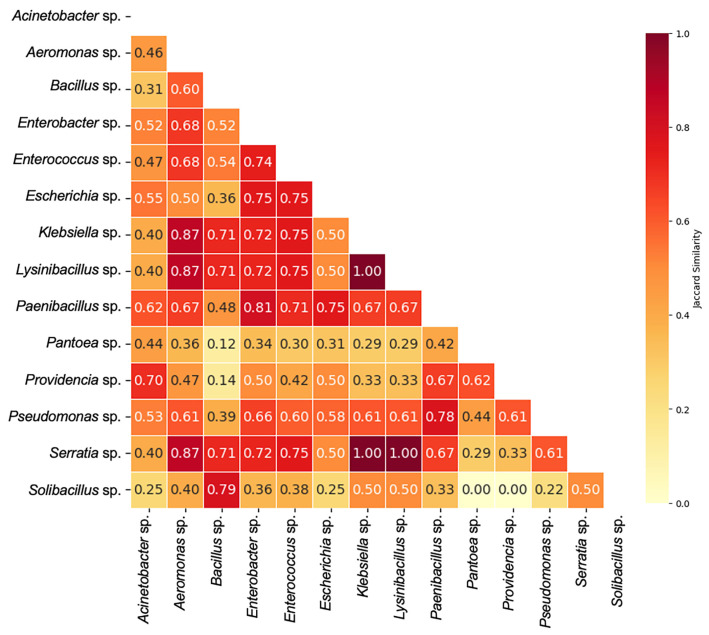
Pairwise similarity of resistance patterns among bacterial genera, calculated using the Jaccard similarity index. Values range from 0 (lowest similarity, light yellow) to 1 (highest similarity, dark red).

To compare resistance structures more broadly across genera, pairwise similarity of binary resistance phenotypes was assessed using the Jaccard similarity index aggregated at the genus level (Figure 4). The mean pairwise similarity across genera was 0.552, indicating a moderate overlap in resistance profiles within the isolate collection. Approximately 26% of genus pairs showed high similarity (>0.7), whereas 24% exhibited low similarity (≤0.4).

A cluster of highly similar resistance profiles was observed among *Klebsiella*, *Lysinibacillus*, and *Serratia*, which exhibited identical binary resistance patterns (Jaccard similarity = 1.00). *Aeromonas* also showed high similarity (>0.87) with this cluster. Several additional genera, including *Enterobacter*, *Enterococcus*, and *Paenibacillus*, displayed moderate similarity (>0.6) to other ARB genera. In contrast, *Solibacillus* exhibited the lowest similarity (0.344), indicating a relatively unique resistance profile compared to other genera.

The results showed a higher prevalence of ampicillin and streptomycin resistance, consistent with previous studies suggesting the widespread presence of beta-lactamase and aminoglycoside resistance genes in farmland ecosystems [55,56]. Antibiotic resistance profiles in natural agricultural fields differ from those with direct antibiotic application. Common resistances, such as tetracycline resistance, may be absent depending on the fitness advantage of the resistance genes without antibiotic pressure [53,57]. Furthermore, it has been reported that the *bla* beta-lactamase gene frequently coexists with additional resistance determinants, such as *add* and *aac* genes (encoding aminoglycoside-modifying enzymes), or *cml* genes (encoding chloramphenicol efflux proteins) [58]. The common resistance frequencies observed for ampicillin, streptomycin, and chloramphenicol in our samples may therefore reflect the dissemination of these antimicrobial resistance determinants.

The findings further identified bacterial genera sharing common resistance profiles across multiple antibiotics. These genera play an important role as bridging taxa within environmental resistance networks and facilitate AMR spread. Multidrug-resistance patterns detected in *Klebsiella* spp. and *Serratia* spp. are ecologically significant, as these opportunistic pathogens are known for their strong capacity to acquire and disseminate antibiotic resistance genes (ARG) [59,60]. Under stress conditions, co-selection of these strains was reported to occur through horizontal gene transfer of ARGs [61]. Antibiotic resistance mechanisms may be broadly distributed across phylogenetically distant species or predominantly associated with specific taxa [62]. In addition to intrinsic resistance in certain genera, similarities in resistance patterns among taxonomically distinct genera may reflect acquired resistance mechanisms [63]. The occurrence of similar resistance patterns among distant genera, together with significant genus-level differences in our findings, suggests the presence of multiple antibiotic resistance genes rather than the widespread dissemination of a single acquired resistance determinant. Observed variation among isolates within the same genus further indicates that resistance phenotypes cannot be reliably inferred. Further research incorporating metagenomic analyses is necessary to identify resistance genes and assess their potential for mobility.

### 3.3. Antagonistic Activity of Yeast Isolates

**Figure 5:**
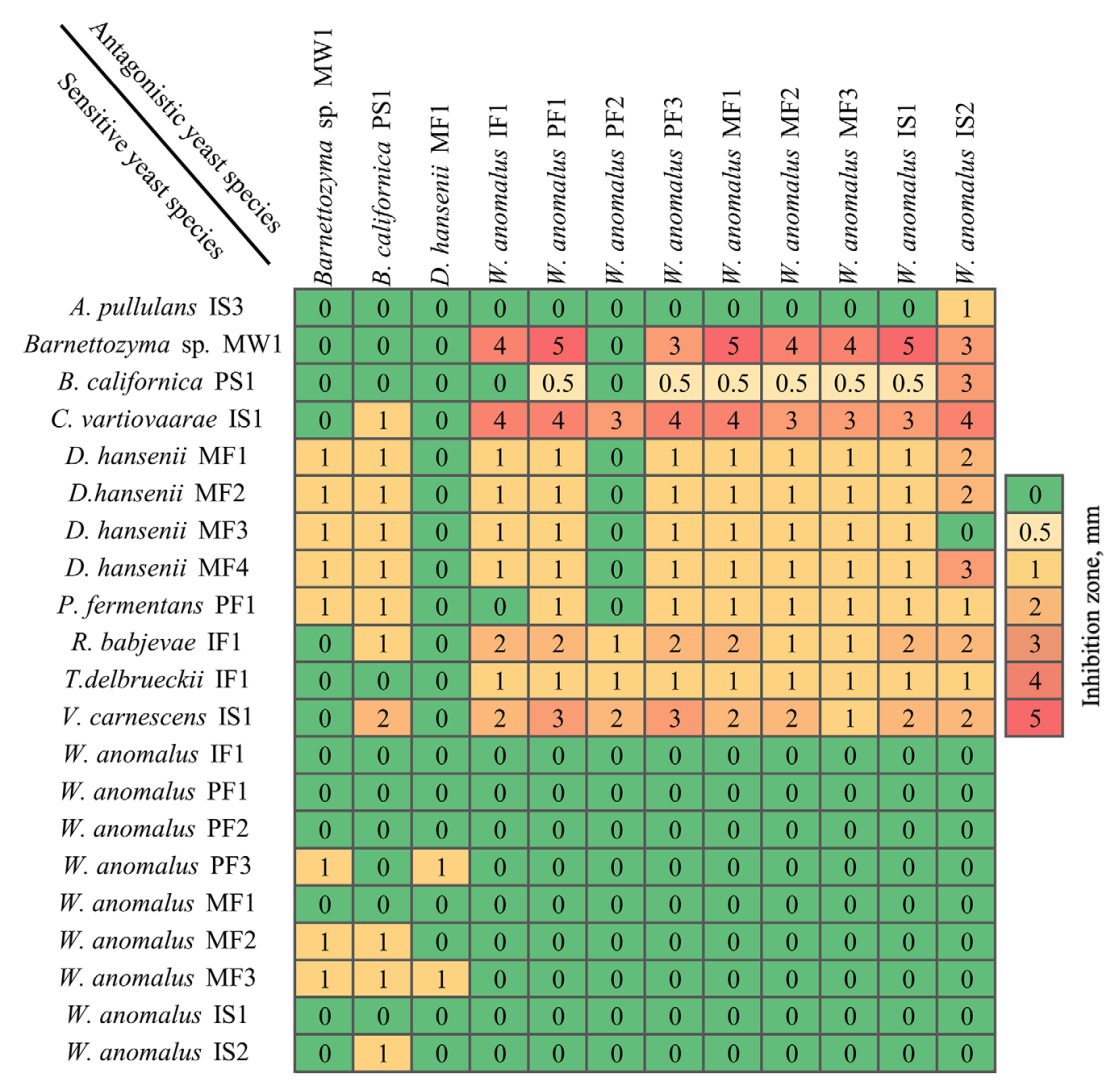
Heatmap of antagonistic activity among yeast isolates. The numbers indicate the diameters (mm) of the inhibition zones surrounding the colonies after incubation, with the largest zone represented in red and the smallest in green.

Antagonistic interactions among the 25 yeast isolates were evaluated using inhibition assays. Each isolate was tested against the remaining members of the collection (600 combinations) at three pH conditions (pH 4.0, 4.8, and 5.6), since previous studies have shown that antagonistic activity varies depending on the acidity of the medium [64]. The strongest and most frequent inhibition was observed at pH 4.8, and subsequent analyses therefore focused on this condition. Under these conditions, 111 of the yeast-yeast pairings (18.5%) resulted in measurable inhibition zones (Figure 5). Inhibition zone diameters ranged from weak inhibition (0.5–1 mm) to strong inhibition exceeding 4 mm. In total, 12 yeast isolates demonstrated inhibitory activity against at least one target yeast strain.

Among the tested isolates, *W. anomalus* strains IF1, PF1, PF3, MF1, MF2, MF3, IS1, and IS2 demonstrated the strongest antagonistic activity, exceeding 4 mm against *Barnettozyma* spp. MW1, and/or *C. vartiovaarae* IS1. Moderate inhibition (approximately 1–3 mm lysis zones) was observed against *Vishniacozyma carnescens* IS1 and *Rhodotorula babjevae* IF1. Among the non-*Wickerhamomyces* strains tested, *D. hansenii* MF1 displayed limited antagonistic capacity, causing only minor inhibition against *W. anomalus* PF3 and MF3. *Barnettozyma* spp. MW1 and *Barnettozyma californica* PS1 demonstrated minimal inhibitory activity overall.

The potent and broad-spectrum activity of *W. anomalus* strains observed here aligns with the literature identifying this species as a promising biocontrol agent [65,66]. *W. anomalus* is known to produce broad-spectrum killer toxins or lytic enzymes (e.g., exo-β-1,3-glucanases) that degrade cell walls of susceptible fungi and other antimicrobial compounds capable of inhibiting competing yeasts and fungi in culture [67]. The weak antagonism exhibited by *D. hansenii* isolates contrasts with its reported biocontrol activity, but its activity is highly strain-dependent and often weaker compared to *W. anomalus* [68,69]. The isolates showing the strongest antagonistic activity were selected for further evaluation against antibiotic-resistant bacterial strains.

### 3.4. Antibacterial Activity Against Antibiotic-Resistant Bacteria

The antibacterial activity of yeast isolates exhibiting the highest antagonistic activity was further assessed against all antibiotic-resistant bacterial isolates recovered in this study. Antagonistic effects were observed only against *Bacillus* strains (PST1, PFT2, MSS1, MFS3), while no inhibition was detected against other resistant bacterial genera. Therefore, Table 1 presents only yeast-bacteria combinations in which susceptibility was detected. In total, 45 inhibition zones were recorded, ranging from 0.49 mm to 4.27 mm in diameter, with an overall mean of 2.01 ± 0.20 mm. Antibacterial activity varied both across yeast isolates and among *Bacillus* strains. Among the tested bacteria, PST1 and PFT2 were the most susceptible, consistently exhibiting larger inhibition zones, whereas MSS1 showed lower overall sensitivity.

*W. anomalus* isolates exhibited the strongest and most consistent antibacterial activity across all four *Bacillus* spp. strains tested. Strain MF1 demonstrated broad inhibition, producing large zones against *Bacillus* PST1 (3.54 ± 0.37 mm), PFT2 (3.44 ± 0.25 mm), MSS1 (1.94 ± 0.23 mm), and MFS3 (3.85 ± 0.16 mm). Similarly, *W. anomalus* IS1 showed strong activity against all four *Bacillus* spp. strains, with inhibition zones ranging from 1.35 mm to 3.74 mm. Other *W. anomalus* strains MF2, MF3, and IS2 exhibited lower activity against all tested strains. The largest inhibition zone observed in the study (4.27 ± 0.51 mm) was produced by *B. californica* PF2 isolate against MFS3, while its activity against other *Bacillus* spp. strains was moderate. *B. californica* IF1 and PF3 inhibited all tested antibiotic-resistant *Bacillus* spp. although the inhibition zones were generally smaller. strains. In comparison to tested yeast isolates, *B. californica* PS1, and *Barnettozyma* spp. MW1 showed weaker and strain-dependent antibacterial activity. *Barnettozyma* spp. MW1 inhibited PST1 (1.27 ± 0.20 mm), PFT2 (1.32 ± 0.36 mm), and MSS1 (0.49 ± 0.06 mm), but showed no activity against MFS3. Similarly, *B. californica* PS1 showed moderate activity against PST1 and PFT2 but no detectable inhibition against MSS1 and MFS3 strains. Antibacterial activity of yeast has been reported previously, particularly *W. anomalus*, which can inhibit microorganisms through the production of antimicrobial peptides, killer toxins (mycocins), hydrolytic enzymes, and other bioactive compounds, disrupting microbial cell membranes and/or interfering with essential cellular processes [70]. These metabolites have been shown to inhibit a range of microorganisms, including Gram-positive bacteria such as *Staphylococcus aureus* and *Bacillus cereus*, as well as certain Gram-negative pathogens such as multidrug-resistant *Acinetobacter baumannii* and *Klebsiella pneumoniae* [65,71,72,73]. The outer membrane of Gram-negative bacteria may limit the penetration of these antimicrobial compounds, which could explain the absence of inhibition in our study for non-*Bacillus* isolates. Although inhibition of the tested *Acinetobacter* strains was not observed in the present study, other studies have reported the susceptibility of multidrug-resistant *Acinetobacter baumannii* to mycocins produced by *W. anomalus* [73]. The limited inhibition towards non-*Bacillus* isolates demonstrates that the antagonistic activity of the tested yeasts is selective and taxon-dependent, rather than universally effective against ARB. Beyond antibacterial effects, *W. anomalus* also showed strong antifungal activity. This yeast species is known for the secretion of mycocins, with β-1,3-glucanase activity that targets cell wall components in susceptible yeasts, leading to cell lysis [74]. These toxins, together with volatile compounds, have demonstrated inhibitory activity against *Candida* spp. and other fungi [67].

The inhibition efficiency observed in this study under laboratory conditions indicates biologically meaningful effects and suggests that antagonistic yeasts may contribute to the natural suppression of specific antibiotic-resistant bacterial populations, particularly *Bacillus* spp. in agricultural ecosystems. Correspondence analysis demonstrated that *Bacillus* and *Wickerhamomyces* were associated with the same farmland compartments, particularly soil and feed. Such spatial co-occurrence may suggest underlying microbial interactions influencing niche structure and ecological impact. In soil and organic substrates, *Bacillus* species are common members of the microbial community, often forming durable endospores that allow survival under harsh conditions [41,42]. The selective inhibition of antibiotic-resistant *Bacillus* spp. by *W. anomalus* suggests that antagonistic yeasts can influence bacterial community structure and contribute to the ecological dynamics of antimicrobial resistance in agricultural ecosystems.

While the ecological relevance of such antagonism in situ remains to be determined, the in vitro inhibition assay applied in this study represents a simplified experimental system that does not fully reflect the complexity of natural environments, where nutrient availability, physicochemical conditions, and interactions within microbial communities may influence antagonistic activity and shape the persistence of antibiotic resistance in agricultural environments. Consequently, the absence of inhibition against certain bacterial strains, particularly Gram-negative bacteria, should not be interpreted as definitive evidence that such interactions do not occur under natural environmental conditions. The culture-based approach captures only the cultivable fraction of environmental microbiota, underestimating overall diversity. In the study, sampling was limited to a small number of farmland sites and conducted within a single season. Additionally, antibiotic resistance classification of farmland strains may not be accurate due to the limited availability of standardized breakpoints for environmental isolates. Future work should include metagenomic analyses to identify resistance genes and their mobility, seasonal monitoring to assess temporal dynamics, and molecular characterization of yeast-derived antimicrobial compounds.

This study evaluated the co-selection of antibiotic-resistant bacteria and antagonistic yeasts across pasture compartments. Resistant bacterial isolates were recovered from all habitats, while yeast-mediated inhibition was selective and restricted to specific taxa. These findings indicate compartment-associated patterns and taxon-dependent antagonistic potential rather than uniform suppression of resistant bacteria. In combination with stringent regulations and measures, extensive monitoring of antimicrobial resistance in agricultural fields is needed to identify potential transmission pathways. As tested in this study, further exploration of microbial interactions could facilitate the development of novel biocontrol agents effective against antibiotic-resistant strains. The findings of such environmental screening research are particularly valuable for agricultural experts and for authorities tasked with shaping related rules and regulations. Lastly, education and awareness of responsible antibiotic use remain critical and must be actively promoted among farmers and veterinarians.

Our findings highlight the role of farmland ecosystems as interconnected microbiomes and emphasize the importance of integrated One Health strategies. Practices such as prudent antimicrobial use, improved environmental management, and systematic monitoring across veterinary, environmental, and agricultural domains are critical for sustaining ecosystem health and resilience.

## 4. Conclusions

In this study, distinct habitat-associated distribution patterns were demonstrated among cultivable antibiotic-resistant bacteria and yeasts, reflecting strong habitat-specific structuring within agricultural ecosystems. Cultivable microorganisms, isolated from soil, feed, and freshwater of Lithuanian pastoral farmlands, were assigned to 14 bacterial and 9 yeast genera, with *Bacillus* and *Wickerhamomyces* isolates detected most frequently. The soil supports the highest diversity of antibiotic-resistant bacterial genera, while feed samples contain the most diverse yeast communities. The antimicrobial resistance of bacteria and the antagonistic activity of yeasts distributed in Lithuanian pastoral farmlands were investigated using an in vitro approach. Ampicillin exhibited the highest resistance frequency among the recovered bacterial isolates, whereas tetracycline resistance was the least prevalent. The frequent occurrence of multidrug-resistant bacteria across compartments showed the natural antimicrobial resistance, even under low-input conditions. Several yeast strains, particularly *W. anomalus,* exhibited pronounced antagonistic activity against both sensitive yeasts and antibiotic-resistant *Bacillus* spp., suggesting that natural microbial antagonism may influence bacterial community structure in agricultural environments. Together, these findings highlight the ecological interconnectedness of soil, feed, and freshwater habitats and emphasize the importance of considering microbial interactions when assessing environmental sources of antimicrobial resistance. Although restricted to cultivable microorganisms, comparisons among soil, feed, and freshwater samples showed differences in the genera identified, their resistance patterns, and yeast antagonistic activity. These results provide a basis for integrating culture-based functional screening into AMR monitoring and for designing follow-up studies that test the role of microbial interactions in managing resistance within agricultural systems.

## Figures and Tables

**Table 1:** Antagonistic activity of yeast isolates against ARB, expressed as mean lysis zone diameter (mm ± SD).

Yeast Strains		Antibiotic-Resistant *Bacillus* spp. Strains			
		PST1	PFT2	MSS1	MFS3
***Barnettozyma*** **sp.**	MW1	1.27 ± 0.20	1.32 ± 0.36	0.49 ± 0.06	0
***Barnettozyma californica***	PS1	1.52 ± 0.11	1.68 ± 0.20	0	0
***Wickerhamomyces anomalus***	IF1	1.88 ± 0.17	1.87 ± 0.16	0.53 ± 0.07	0.71 ± 0.23
	PF1	1.99 ± 0.14	1.91 ± 0.42	1.15 ± 0.23	2.31 ± 0.18
	PF2	2.95 ± 0.12	2.94 ± 0.30	1.80 ± 0.20	4.27 ± 0.51
	PF3	2.29 ± 0.12	1.74 ± 0.13	0.87 ± 0.13	1.82 ± 0.22
	MF1	3.54 ± 0.37	3.44 ± 0.25	1.94 ± 0.23	3.85 ± 0.16
	MF2	2.65 ± 0.06	2.65 ± 0.14	1.10 ± 0.40	1.98 ± 0.10
	MF3	2.32 ± 0.08	2.03 ± 0.06	0.51 ± 0.13	1.60 ± 0.06
	IS1	3.74 ± 0.38	2.72 ± 0.13	1.35 ± 0.44	2.83 ± 0.31
	IS2	1.99 ± 0.31	2.16 ± 0.17	0.62 ± 0.12	1.93 ± 0.13

## Data Availability

The original contributions presented in this study are included in the article/Supplementary Materials. Further inquiries can be directed to the corresponding authors.

## Supplementary Materials

The following supporting information can be downloaded at: https://www.mdpi.com/article/10.3390/microorganisms14050991/s1, Table S1: MIC interpretive criteria used for classification of antibiotic resistance in bacterial genera, based on CLSI guidelines and adapted for environmental isolates; Figure S1: Representative colony morphology of bacterial (A) and yeast (B) isolates obtained in this study.
