# Supplementary material for: Cross-Kingdom Antagonistic Interactions Between Environmental Antibiotic-Resistant Bacteria and Yeasts in Pastoral Ecosystems
Source: Microorganisms. 2026 Apr 28;14(5):991. doi: 10.3390/microorganisms14050991 (PMC13209842; doi:10.3390/microorganisms14050991)
Supplement: Supplementary file 1 [file microorganisms-14-00991-s001.zip › TableS1.pdf]

Table S1. MIC interpretive criteria used for classification of antibiotic resistance in bacterial genera, based on CLSI guidelines and adapted for environmental isolates.

Genera	Antibiotic	CLSI Breakpoint (µg/mL)
Acinetobacter	Ampicillin	128≤R
	Streptomycin	S≤4-16≤R
	Tetracycline	S≤4-16≤R
	Chloramphenicol	S≤8-32≤R
Aeromonas	Ampicillin	128≤R
	Streptomycin	S≤4-16≤R
	Tetracycline	S≤4-16≤R
	Chloramphenicol	S≤8-32≤R
Bacillus	Ampicillin	S≤0.25-0.5≤R
	Streptomycin	S≤4-16≤R
	Tetracycline	S≤4-16≤R
	Chloramphenicol	S≤8-32≤R
Enterobacter	Ampicillin	S≤8-32≤R
	Streptomycin	S≤4-16≤R
	Tetracycline	S≤4-16≤R
	Chloramphenicol	S≤8-32≤R
Enterococcus	Ampicillin	S≤8-16≤R
	Streptomycin	128≤R
	Tetracycline	S≤4-16≤R
	Chloramphenicol	S≤8-32≤R
Escherichia	Ampicillin	S≤8-32≤R
	Streptomycin	S≤4-16≤R
	Tetracycline	S≤4-16≤R
	Chloramphenicol	S≤8-32≤R
Klebsiella	Ampicillin	S≤8-32≤R
	Streptomycin	S≤4-16≤R
	Tetracycline	S≤4-16≤R
	Chloramphenicol	S≤8-32≤R
Lysinibacillus	Ampicillin	S≤0.25-0.5≤R
	Streptomycin	S≤4-16≤R
	Tetracycline	S≤4-16≤R
	Chloramphenicol	S≤8-32≤R
Paenibacillus	Ampicillin	S≤0.25-0.5≤R
	Streptomycin	S≤4-16≤R
	Tetracycline	S≤4-16≤R
	Chloramphenicol	S≤8-32≤R
Pantoea	Ampicillin	S≤8-32≤R
	Streptomycin	S≤4-16≤R
	Tetracycline	S≤4-16≤R
	Chloramphenicol	S≤8-32≤R
Providencia	Ampicillin	S≤8-32≤R
	Streptomycin	S≤4-16≤R
	Tetracycline	S≤4-16≤R
	Chloramphenicol	S≤8-32≤R
Pseudomonas	Ampicillin	128≤R
	Streptomycin	S≤4-16≤R
	Tetracycline	S≤4-16≤R
	Chloramphenicol	S≤8-32≤R
Solibacillus	Ampicillin	S≤0.25-0.5≤R
	Streptomycin	S≤4-16≤R
	Tetracycline	S≤4-16≤R
	Chloramphenicol	S≤8-32≤R
Serratia	Ampicillin	S≤8-32≤R
	Streptomycin	S≤4-16≤R
	Tetracycline	S≤4-16≤R
	Chloramphenicol	S≤8-32≤R
