# Supplementary figures and images for: Cross-Kingdom Antagonistic Interactions Between Environmental Antibiotic-Resistant Bacteria and Yeasts in Pastoral Ecosystems
Source: Microorganisms. 2026 Apr 28;14(5):991. doi: 10.3390/microorganisms14050991 (PMC13209842; doi:10.3390/microorganisms14050991)

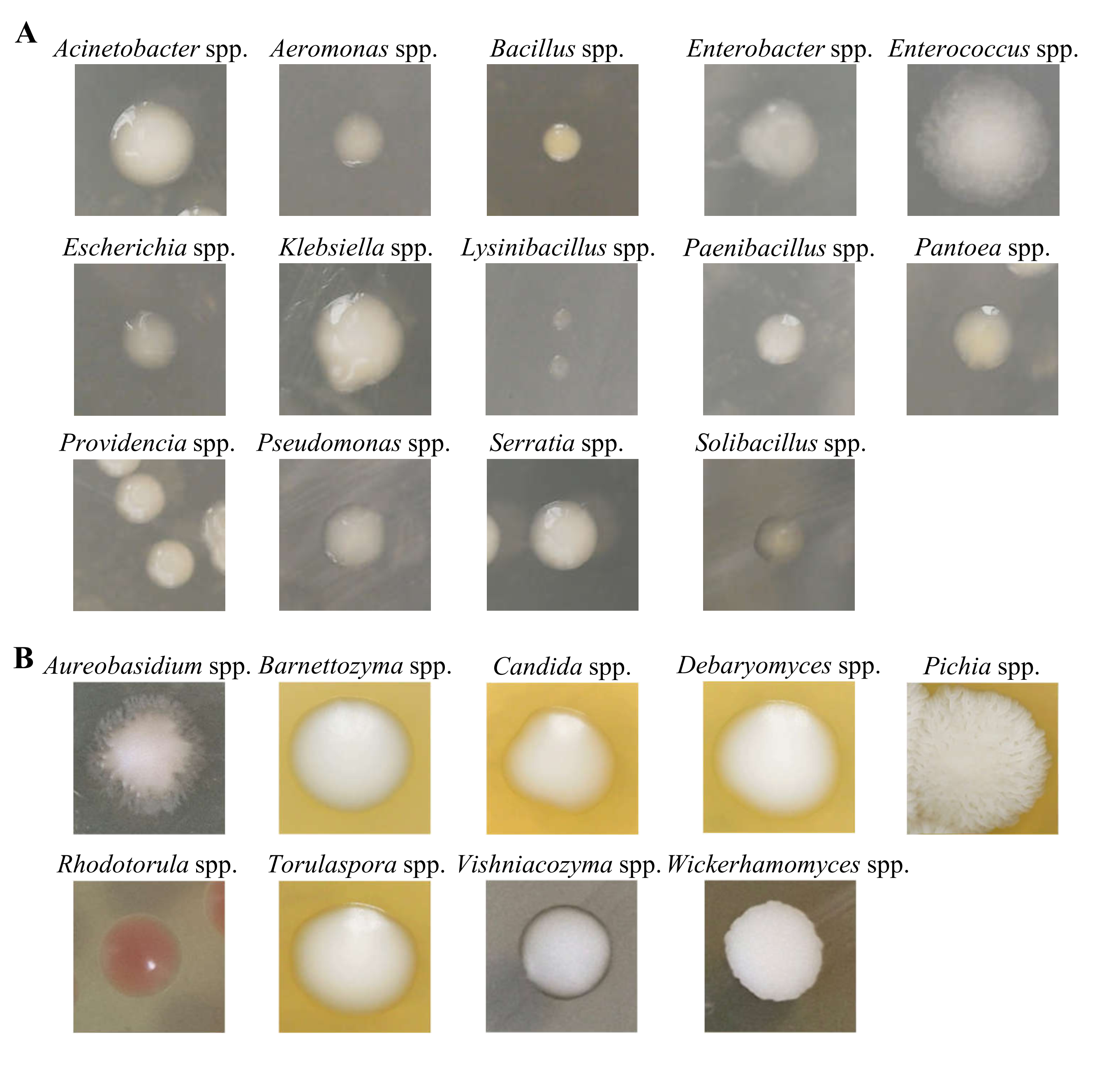

Supplement: Supplementary file 1 [file microorganisms-14-00991-s001.zip › FigureS1.tif]
